# Eyes on you: Ensuring empathic accuracy or signalling empathy?

**DOI:** 10.1002/ijop.12862

**Published:** 2022-06-13

**Authors:** Mirjam C.M. Wever, Lisanne A. E. M. van Houtum, Loes H. C. Janssen, Iris M. Spruit, Marieke S. Tollenaar, Marije aan het Rot, Bernet M. Elzinga

**Affiliations:** ^1^ Institute of Psychology Leiden University Leiden The Netherlands; ^2^ Leiden Institute for Brain and Cognition Leiden The Netherlands; ^3^ Department of Psychology University of Groningen Groningen The Netherlands; ^4^ School of Behavioural and Cognitive Neurosciences University of Groningen Groningen The Netherlands

**Keywords:** Empathic accuracy, Eye gaze, Empathic concern, Perspective taking, Social functioning

## Abstract

The eye region is thought to play an important role in the ability to accurately infer others' feelings, or empathic accuracy (EA), which is an important skill for social interaction. However, most past studies used static pictures, including only visual information, and knowledge about the contribution of the eye region to EA when visual information is presented together with verbal content is lacking. We therefore examined whether eye gazing contributes to EA during videos of emotional autobiographical stories including both visual and verbal content. One hundred seven perceivers watched videos of targets talking about positive and negative life events and continuously rated the targets' feelings during the videos. Simultaneously, perceivers' eyes were tracked. After each video, perceivers reported on their feelings and the extent to which they empathized with and took the perspective of the targets. In contrast to studies using static pictures, we found that gazing to the eyes of targets during the videos did not significantly contribute to EA. At the same time, results on the association between the amount of gaze towards the eye region of targets and perceivers' state and trait empathy ratings suggest that eye gazing might signal empathy and social engagement to others.

The ability to empathize with others is often considered a key ingredient for successful social interactions. However, the *accuracy* of inferring another's thoughts and feelings, also referred to as empathic accuracy (EA), is at least equally important (Ickes & Hodges, 2013; Zaki et al., 2008; Zaki et al., 2009). Several studies have emphasised the importance of the eye region for inferring the internal states of others (Baron‐Cohen et al., 1997; Buchan et al., 2007; Eisenbarth & Alpers, 2011; Hall et al., 2010; Zaki et al., 2009). A task that emphasises the importance of the eyes to infer the internal states of others is the reading the mind in the eyes task (Baron‐Cohen et al., 1997). Numerous studies using this task have shown that a person's eye region contains sufficient information to identify complex mental states (Baron‐Cohen et al., 1997). Moreover, the eye region automatically attracts and maintains attention, especially under emotional circumstances (Cowan et al., 2014) and people are generally found to gaze more towards the eyes of others compared to other facial features (e.g., mouth, nose or cheeks) (Buchan et al., 2007; Eisenbarth & Alpers, 2011; Hall et al., 2010).

Notably, however, EA in real life usually entails a mixture of visual and verbal information about the social situation involved. Nonetheless, most studies into the role of eye gaze in emotion recognition made use of static pictures including only visual information. This limits the generalizability to real‐life social interactions. Moreover, when the specific contribution of visual and verbal information of targets to the EA of perceivers is examined, verbal information has been found to contribute more to EA than visual information, but a combination of both produces the highest EA (Zaki et al., 2009). Furthermore, static pictures of clear facial emotional expressions do not capture how our emotions are expressed in daily life, which can be much more subtle and ambiguous. So, while gazing to the eye region of others is beneficial under circumstances in which the informational source is limited to visual input, the added value of eye gazing when combined with verbal information is still unclear. So far, two studies have examined the association between eye gazing and trait empathy using a similar set of dynamic stimuli with both visual and verbal content (Cowan et al., 2014; Martínez‐Velázquez et al., 2020). In these studies, people gazed more towards the eyes of others in emotional versus neutral video and the amount of eye gazing was positively associated with people's trait empathy levels. Building on these studies it is of interest to examine whether gazing towards the eyes of others also contributes to EA when visual information is presented together with verbal content.

Gazing to the eyes of others may be especially helpful in situations in which social cues are ambivalent. Happy faces are quickly recognised, and eye fixations are mostly directed to the mouth region, probably because a smile on the mouth is a clear and distinctive feature of happiness (Calvo et al., 2008; Eisenbarth & Alpers, 2011). Sad expressions, in contrast, generally include less distinctive facial features and people fixate more towards the eye region during these expressions, possibly to search for additional emotion cues (Bombari et al., 2013; Eisenbarth & Alpers, 2011). As such, looking at the eye region of others might contribute more strongly to EA during negative versus positive emotional situations. Besides, the eye region might be particularly informative when facial expressions are rather subtle or ambiguous (Baron‐Cohen et al., 1997; Vaidya et al., 2014). As facial expressions are generally less pronounced in less emotionally expressive persons, looking the eye region of others might contribute more to EA when these “others” are less emotionally expressive.

In the present study, we aimed to determine whether gazing to the eyes of others contributes to EA when verbal content is present as well. In addition, we examined whether this was dependent on the valence of the story content and targets' emotional expressivity. All measures and hypotheses of this study were preregistered at Open Science Framework prior to data analyses (https://osf.io/qxdv9/). We hypothesized that (a) perceivers who look more in the eye region of targets show higher EA, (b) the amount of gaze towards the eyes of others is a stronger predictor of EA in negative versus positive videos, and (c) the amount of gaze towards the eyes of others is a stronger predictor of EA when targets are less emotionally expressive.

## METHOD

### Participants

Data were collected in the context of the RE‐PAIR study: “Relations and Emotions in Parent‐Adolescent Interaction Research”. This study examines the relation between parent‐adolescent interactions and adolescent mood. Families were eligible for inclusion if the adolescent and at least one of the parents were willing to participate and had a good command of the Dutch language. Further inclusion criteria were applied to the adolescents and can be found in Supplement S1. There were no additional in‐ or exclusion criteria for the parents.

The present study includes data of the parents of adolescents without psychopathology (*n* = 150); subsequently referred to as participants or as *perceivers*. Data of 43 participants were excluded. Complete task data of five participants were lost due to technical problems with the task, and gaze data of 38 participants were missing due to unsuccessful calibration of the eye tracker. Reasons for the failure of calibration procedures were sight deficiencies, participants wearing glasses, or participants having light‐coloured eyes, which are all confirmed in prior studies to affect gaze data quality (Kammerer, 2009; Nyström et al., 2013). Of note, the EA task was embedded in a larger study for which the in‐ and exclusion criteria were not explicitly tailored to inclusion for eye tracking.

For 13 participants eye gaze data were missing on one or more videos (42 videos in the total, range: 1–8) and 11 participants had <70% valid gaze data on one or more videos (37 videos in the total, range: 1–8). In addition, 17 participants of the final sample were missing continuous EA ratings on one or more videos (37 videos in the total, range: 1–5) due to technical problems during the task or inadequate use of the dial. This resulted in a final sample of 107 participants with 981 videos in the total for the analyses (out of 1070, 8.3% missing data), including 48 males (45%, *M*_*age*_ = 50 years, *SD* = 5.97) and 59 females (*M*_*age*_ = 47 years, *SD* = 4.75). The final sample (*n* = 107) was representative for the total number of participants that performed the EA task (*n* = 150) as they did not significantly differ on age, gender, trait empathy, autism spectrum traits, and intellectual functioning.

The study was approved by the medical ethical committee of the Leiden University Medical Centre (LUMC) (P17.241) and was performed in accordance with the declaration of Helsinki and the Dutch Medical Research Involving Human Subjects Act (WMO). All participants provided written informed consent at the start of all study visits and were blind to the hypotheses of the present study.

### Procedure

Families were recruited via public places and social media. Parents and adolescents were briefed about the study and underwent a comprehensive telephone screening during which family circumstances and verbal informed consent were discussed. When found eligible for participation, families were invited for a lab visit to the Leiden University Treatment and Expertise Centre (LUBEC) in Leiden. Two weeks prior to the appointment participants were asked to fill out an online questionnaire battery that included questions about demographics and clinical and cognitive constructs (see Measures and materials). During the lab visit, families performed parent‐adolescent interaction tasks and filled out additional questionnaires, parents were screened for psychopathology and intellectual abilities were assessed. Furthermore, parents performed the EA task while eye‐tracking measures were taken, which is the focus of the present study.^1^

### Measures and materials

#### Empathic accuracy task

Similar to the English task (Zaki et al., 2008), the Dutch task developed by Aan het Rot and Hogenelst (2014) includes dynamic stimuli of various target people who are telling both positive/happy (e.g., celebrating a birthday with friends) and negative/sad (e.g., a friend died of a brain haemorrhage) emotional autobiographical stories. Within 30 minutes after the stories of the targets were videotaped, the targets watched their personal recordings and continuously rated how they felt in their videos by using a dial. The dial included a Likert scale ranging from 1 = extremely negative to 9 = extremely positive. Additional information about task development can be found in aan het Rot and Hogenelst (2014).

The targets varied in their self‐reported emotional expressivity as assessed with the Berkeley Expressivity Questionnaire (BEQ; Gross and John (1997)). The BEQ consists of 16 items that are answered on a Likert scale from 1 (strongly disagree) to 7 (strongly agree). Mean BEQ scores were calculated by averaging all items and ranged in the present target sample from 3.50–5.97. Higher scores represent higher emotional expressivity. Prior studies reported a significant positive association between BEQ scores and EA (aan het Rot & Hogenelst, 2014; Zaki et al., 2008). Since the original item of targets on the BEQ could not be retrieved the reliability of the scale in the current sample could not be computed. Yet, previous studies found good validity and reliability for the instrument (α = .86) (Kupper et al., 2020), and there is no reason to expect any differences between these studies and the use of the BEQ in the current sample.

The present study includes a shortened version of the EA task with a duration of ±25 minutes instead of the original 50 minutes. This was performed due to time constraints as the task was part of a larger study protocol. The task included a subset of 10 videos, consisting of five positive and five negative autobiographical stories from six distinct targets (three male, three female) that derived from a pilot study in which we tested the feasibility of the EA task in combination with eye tracking (Supplement  S2).

The participants in the present study (perceivers) were asked to watch all 10 videos and instructed to imagine that they were sitting on the other side of the table of the targets while listening to their autobiographical stories. At the same time, they continuously rated how they thought the target was feeling while narrating, using the same dial as the targets used to rate their feelings. Videos were semi‐randomly presented, with no more than two positive or negative videos and no more than two videos with a target of the same gender in a row. Prior to the start of the task, perceivers were presented with a test trial in which the test leader checked correct use of the dial. Prior to the start of each video perceivers were asked to put the dial back to “neutral” to start each trial from the same position. All procedures are in line with previous studies using this task (aan het Rot & Hogenelst, 2014).

A new addition was that after each video the perceivers were asked to report on how well they were able to empathize with (i.e., *state* empathic concern) and put themselves in the shoes (i.e., *state* perspective taking) of the target. Also, perceivers rated how happy, sad, relaxed and irritated they felt after each video. All questions were rated on a 7‐point Likert scale ranging from 1 = not at all to 7 = very much.

Stimulus presentation and simultaneous eye movement recordings were conducted using E‐Prime 2.0 software with the E‐Prime Extension for Tobii package (Psychology Software Tools, Pittsburgh, PA, United States). The screen resolution was 1920 × 1080 pixels and videos were presented on the screen in 960 × 540 pixels.

#### Eye tracking

Eye movements were recorded with a portable Tobii Pro X3‐120 eye‐tracker sampling at 120 Hz. Prior to the start of the task perceivers were asked to place their head in a chin rest to prevent head movement during the recording and the distance to the screen was set at 60 cm. Perceivers' eyes were calibrated using a 9‐point calibration grid and calibration results were visually inspected and accepted if quality was approved. In case of missing calibration points or poor calibration quality, the procedure was repeated for a maximum of three attempts after which the quality was unlikely to further improve. The EA task started directly after the calibration procedure and gaze data was recorded until the task was finished.

#### Trait empathy

To assess trait empathy perceivers filled out the empathic concern (EC) and perspective taking (PT) subscales of the interpersonal reactivity index (IRI) prior to the start of the lab visit (Davis, 1980; De Corte et al., 2007). EC includes the reported tendency to experience feelings of sympathy and compassion for unfortunate others and PT includes the reported tendency to spontaneously adopt the psychological point of view of others in everyday life. Both subscales include 7 items and are answered on a 5‐point Likert scale ranging from 0 (does not describe me well) to 4 (describes me very well). Sum scores of each subscale were calculated by adding up the items (range in the present sample was 7–28 for EC and 6–27 for PT). Higher scores represent higher trait empathy levels. The validity and reliability of the Dutch IRI has been established (De Corte et al., 2007) and the internal consistencies of the subscales in the present sample were acceptable (α = 0.75 for both).

#### Intellectual functioning

Intellectual functioning was assessed with two subtests of the Dutch Wechsler Adult Intelligence Scale IV (WAIS‐IV‐NL; Wechsler (2012)); block design (perceptual organisation skills) and vocabulary (verbal skills). Individual raw scores were translated into norm scores based on age and were averaged to calculate the estimated intellectual functioning measure per individual. This measure was included as covariate in the analyses to control for individual differences in intellectual functioning. Validity of this subtest dyad with the original full‐scale IQ has been established (Girard et al., 2015).

### Data analyses

#### Preprocessing

Preprocessing of the raw data from the EA task was similar to aan het Rot and Hogenelst (2014), with raw continuous ratings from perceivers and targets being preprocessed into an EA measure per video in SAS 9.3 for Windows (SAS, Cary, NC). For data reduction purposes, ratings from perceivers and targets were averaged across 5‐second periods. The last 5 seconds of all ratings were discarded, because it includes the return of the dial to the “neutral” position before the end of each video. Subsequently, first‐order autocorrelations were removed from the continuous ratings using the Yule–Walker method. For each video, we correlated perceiver ratings of the target's feelings and target ratings of their own feelings, resulting in a correlation coefficient *r* that defined the perceiver's raw EA score per video. Raw EA scores underwent a Fisher *z* transformation prior to further analyses.

See Supplement S3 for more details on the pre‐processing of raw eye‐tracking data into measures of eye gaze per perceiver per video. The primary eye gaze measure is the percentage dwell time within the defined areas of interest (AOIs; i.e., eyes, mouth, and face as a whole) per video, as part of the total video duration, in which dwell time is defined as the total amount of time spent looking within an AOI and includes all types of eye movements. The percentage dwell time within the face and mouth AOI were described to identify to what extent perceivers gazed towards the face and mouth of the targets in addition to their eye region. Dwell time is interpreted as the level of interest in an AOI, with greater dwell times indicating greater levels of interest.

#### Statistical analyses

Means and standard deviations of the EA task and the self‐report ratings per video and valence category (i.e., positive or negative) were calculated. In addition, the average percentage dwell time for each AOI (i.e., eyes, mouth and face) per valence category and video were assessed.

The effects of our hypothesized predictors on EA were tested in R‐3.6.1 (R Core Team, 2013), using generalised linear mixed regression models with a multi‐level, within‐subject design. We used lme4 for multilevel analyses with maximum likelihood (Bates et al., 2012) and ggplot2 for figures (Wickham et al., 2016). The dependent variable EA has been repeatedly measured and EA observations per video (level 1) were nested within perceivers (level 2). Predictor variables that act upon the perceiver‐level were the percentage dwell time within the eye region and trait EC and PT. Predictor variables that act upon the target‐level (level 1) were the target expressivity and valence of the videos.

First, we ran correlations between all predictor variables (i.e., percentage dwell time within the eye region, target expressivity, valence and trait EC and PT) and the outcome variable EA. Thereafter, we tested the validity of the task by assessing the influence of target expressivity, valence, and trait EC and PT on EA using generalised linear mixed regression models.

Subsequently, we tested our main hypotheses about the influence of the percentage dwell time within the eye region of targets on EA (hypothesis 1) and whether this interacts with the valence of the videos (hypothesis 2) or target expressivity (hypothesis 3) in two separate two‐way interactions. Exploratively, we also tested a three‐way interaction between the percentage dwell time within the eye region of targets, target expressivity, and valence on EA. In case of significant interactions, we broke down the interaction into simple contrasts using Bonferroni‐corrected post hoc tests.

To check whether results are not driven by differences in age, gender and intellectual functioning of perceivers, we performed additional analyses in which we statistically controlled for these variables. Significance was set at *p* < .05 (two‐tailed) and Cohen's *d* effect sizes were calculated for significant effects.

## RESULTS

### Task descriptives

**Figure 1:**
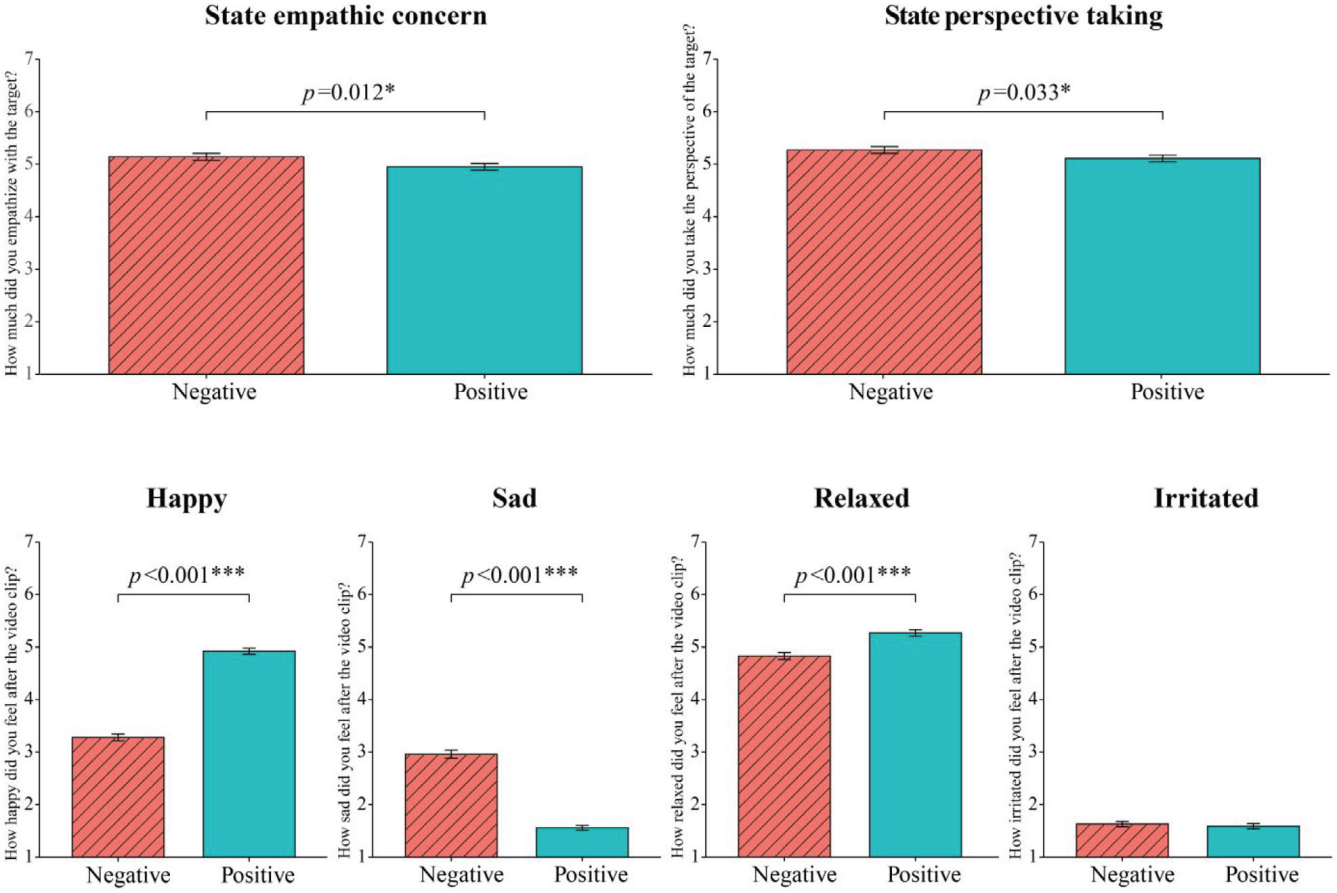
Mean individual ratings of perceivers, plotted for negative and positive videos, rated on a 7‐point Likert scale (1 = not at all, 7 = very much). Significance was tested with linear mixed model analyses. Error bars represent the standard error of the mean. Significant *p*‐values < .05 were indicated by *, *p* < .01 by **, and *p* < .001 by ***.

See Table 1 for demographic and clinical characteristics. Data derived from individual state empathy ratings after each video revealed that perceivers empathize more with and were better able to take the perspective of the targets during negative versus positive videos (*b* = 0.186, *SE* = 0.07, *t*(872.39) = 2.52, *p* = .012, *d* = 0.17 and *b* = .155, *SE* = 0.07, *t*(871.99) = 2.13, *p* = .033, *d* = 0.14, respectively). In addition, they felt significantly more sad after negative videos (*b* = 1.40, *SE* = 0.07, *t*(874.21) = 18.89, *p* < .001, *d* = 1.28) and more happy (*b* = 1.65, *SE* = 0.07, *t*(870.35) = 22.13, *p* < .001, *d* = 1.50) and relaxed (*b* = .45, *SE* = 0.06, *t*(870.75) = 7.17, *p* < .001, *d* = 0.49) after positive videos. There was no significant difference in irritability between positive and negative videos (*p* = .366) (Figure 1). In addition, perceivers who reported higher trait EC and PT were also better able to empathize with (*b* = .05, *SE* = 0.02, *t*(101.98) = 2.864, *p* = .005, *d* = 0.57) and take the perspective of targets (*b* = .06, *SE* = 0.02, *t*(105.03) = 3.05, *p* = .003, *d* = 0.60) based on the state empathy ratings per perceiver (see Supplements S4–S5).

**TABLE 1:** Demographics and clinical data

	Total	Males	Females	
Mean (*SD*)	n = 107	n = 48	n = 59	Gender differences^a^
Age	48 (5.50)	50 (5.97)	47 (4.75)	.005
Autism spectrum traits^b^	54.25 (10.48)	57.92 (11.01)	51.27 (9.08)	<.001
*Trait empathy* ^c^				
Empathic concern	18.08 (4.92)	15.79 (4.29)	19.95 (4.63)	<.001
Perspective taking	17.27 (4.54)	15.94 (4.67)	18.36 (4.15)	.006
*WAIS‐IV* ^d^				
Block design	10.92 (3.16)	11.73 (3.09)	10.23 (3.09)	.020
Vocabulary	11.68 (2.49)	11.73 (2.60)	11.64 (2.60)	.857
Average score	11.30 (2.15)	11.73 (2.28)	10.93 (1.99)	.071

*Note*:. WAIS‐IV, Wechsler Adult Intelligence Scale IV (Wechsler, 2012).

a *p*‐Values were obtained using independent samples *t*‐test comparisons between males and females.

b Autism spectrum traits were assessed with the 28‐item version of the Autism‐spectrum Quotient (AQ‐short; Hoekstra et al. (2011)).

c Trait empathy was assessed with the Interpersonal Reactivity Index (IRI; De Corte et al. (2007)).

d Data on intellectual functioning (WAIS‐IV) was missing or unreliable for 11 participants resulting in *n* = 96 for this measure.

The mean raw *r* between perceivers' and targets' EA was 0.53 and did not differ between males and females. We ran generalised linear mixed model analyses in which we assessed the influence of valence, target expressivity, and trait EC and PT on EA. As expected, but in contrast to the impact of valence on perceivers' state empathy levels, perceivers were less empathically accurate during negative versus positive videos (*b* = −.46, *SE* = 0.06, *t*(881.9) = −8.25, *p* < .001, *d* = 0.56). Target expressivity and trait EC and PT of perceivers were not significantly associated with perceivers' EA (all *p* ≥ .796). All outcomes remained significant after controlling for age, gender, and intellectual abilities of perceivers in separate analyses.

On average, perceivers gazed for 85.7% (*SD* = 8.65%) of the total duration of the videos towards the faces of targets, indicating that the targets' faces substantially attracted and maintained perceivers' attention. In addition, perceivers gazed on average for 33.38% (*SD* = 18.49%) of the total duration of the videos towards the eye region of the targets. There was no significant difference between males and females in the percentage of dwell time towards the eye region of the targets. Perceivers gazed more into the eyes of others during negative versus positive videos (*b* = 3.77, *SE* = 0.61, *t*(873.75) = −6.15, *p* < .001, *d* = 0.42). In addition, perceivers with higher trait EC and PT gazed significantly more into the eyes of others (trait EC: *b* = 0.73, *SE* = 0.36, *t*(104.96) = 2.02, *p* = .046, *d* = 0.40; trait PT: *b* = 0.79, *SE* = 0.39, *t*(105.65) = 2.03, *p* = .045, *d* = 0.40), independent of the emotional valence of the videos. In line with this, perceivers who gazed more within the eye region of targets during a video reported to empathize more with and were better able to take the perspective of the targets narrating the autobiographical story on a state level (*b* = .70, *SE* = 0.28, *t*(906.47) = 2.47, *p* = .014, *d* = 0.16 and *b* = .67, *SE* = 0.29, *t*(905) = 2.33, *p* = .020, *d* = 0.16, respectively). Target expressivity was not significantly associated with perceivers' dwell time within the eye region of the targets (*p* = .571). On average, there was 9.46% (*SD* = 6.53%) missing gaze data during which participants gazed outside of the computer screen. The amount of missing gaze data was not dependent on the presentation order of the videos in the task. For more details on missing gaze data over the course of the task, see Supplement S9.

In addition to the eye region, perceivers gazed on average for 15.79% (*SD* = 15.14%) of the total duration of the videos to the mouth of the targets. Male and female perceivers did not differ significantly in the percentage dwell time to the mouth of the targets. In addition, valence and trait EC and PT were not significantly associated with the percentage dwell time to the mouth of the targets. However, we found a positive association between emotional expressivity of targets and the percentage dwell time of perceivers to the mouth of targets in the videos, with perceivers gazing more to the mouth region of more (compared to less) expressive targets (*b* = .89, *SE* = 0.2094, *t*(873.56) = 4.25, *p* < .001, *d* = 0.04). See Supplement S4–S8.

### Effects of gaze to the eyes on empathic accuracy

**Figure 2:**
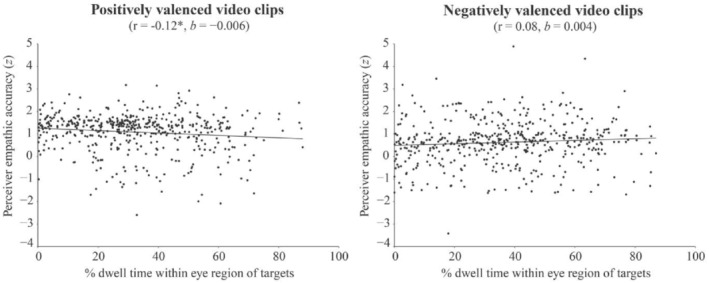
Associations between the percentage dwell time within the eye region of targets and perceivers' EA in positive and negative videos.

With regard to the main focus of our study (hypothesis 1), the percentage dwell time within the eye region of targets was not significantly related to perceivers' EA (*p* = .146). We did find a significant interaction between the percentage dwell time within the eye region of targets and the emotional valence of the videos on perceivers' EA (hypthesis 2) (*b* = −0.01, *SE* = 0.002, *t*(892.78) = −3.33, *p* < .001, *d* = 0.22), although in opposite direction. In contrast with our expectations, there was no significant association between gazing towards the eye region of targets and EA in negative videos, however, perceivers that gazed *more* towards the eye region of the targets during positive videos were somewhat less empathically accurate (Figure 2, Supplement S10). Lastly, there was no significant interaction between the percentage dwell time within the eye region of targets and targets' emotional expressivity on perceivers' EA (hypothesis 3) (*p* = .416).

### Explorative analyses

The finding that perceivers who gazed more towards the eye region of targets during positive videos were less empathically accurate raised the question whether perceivers instead gazed more to the mouth during these videos. Therefore, we additionally explored whether valence also interacted with the percentage dwell time to the mouth of the targets. We examined the interaction between percentage dwell time to the mouth and emotional valence of the videos on perceivers' EA, but this interaction was non‐significant (*p* = .063). Also, there was no significant interaction between the percentage dwell time to the mouth of targets and targets' emotional expressivity on perceivers' EA (*p* = .752) or between the percentage dwell time to the mouth of targets and perceivers' EA in general (*p* = .860).

## DISCUSSION

The present study used a paradigm with high ecological validity to examine whether gazing to the eyes of others contributes to EA during videos of emotionally valenced target stories in which verbal information was also available. First, gazing to the eyes of others did not significantly contribute to EA. Second, however, the emotional valence of the stories did moderate the relation between gazing to the eyes of others and EA: Perceivers who gazed more to the eye region of others during positive target stories were less empathically accurate, whereas this was not found during negative target stories. Third, targets' emotional expressivity was not significantly related to perceivers' EA, nor did it moderate the relation between gazing to the eyes of others and perceivers' EA.

In contrast to our hypotheses, we found that perceivers who gazed more towards the eye region of targets were not more empathically accurate. Moreover, perceivers who gazed less towards the eyes of others during positive videos were even more empathically accurate. Although the importance of the eye region has consistently been demonstrated in studies that solely convey visual input, our findings indicate that the eye region seems to be less informative when visual input is presented in co‐occurrence with verbal information. While prior studies have greatly contributed to our basic understanding of the role of the eye region in social interactions, the current results emphasise the importance of also studying such processes in more ecologically valid settings since conclusions can deviate in important ways.

Our results did not show an effect of target expressivity on perceivers' EA scores, nor did it moderate the relation between gazing to the eyes of targets and perceivers' EA. This was not in line with our hypotheses and prior studies using the Dutch EA task, but might be due to differences in methodology. In the current, shortened, version of the EA task we included six out of 11 targets, which considerably decreased the diversity of target expressivity in the present study. In addition, the BEQ mainly focuses on emotional expressivity in the face of targets, while perceivers also receive verbally expressive informational cues of the targets to base their EA on. It is possible that targets who report to have less expressive faces could still have an expressive tone of voice, which might have revealed information about their internal feelings.

An unexpected finding was that perceivers who considered themselves more empathic, both at trait (EC and PT scales of the IRI) and state level (individual ratings after each video), gazed more towards the eye region of others. In this light, gazing to others' eyes might be a way to express empathy to others, rather than (only) collect (additional) socio‐emotional information about others' internal states. This dovetails with the results of Cowan et al. (2014) and Martínez‐Velázquez et al. (2020), who interpreted the increased gazing towards the eye region of others as enhanced social engagement. Moreover, looking at the eyes of a conversational partner while listening was found to signal interest and affiliation (Breil & Böckler, 2021).

We found that perceivers gazed more towards the eye region of others during negative versus positive videos. In addition, individual ratings of perceivers' state empathy showed that they were better able to empathize with and take the perspective of targets in negative versus positive videos. This is in accordance with the possible signalling function of eye gaze, suggesting that people might have a natural tendency to empathize with and gaze more to the eyes of others during negative versus positive emotional situations. This effect may have been emphasised by the stimuli duration (±1–2 minutes), as empathic feelings are particularly induced after prolonged presentation duration (Regenbogen et al., 2012). It is of note, however, that participants were instead less empathically accurate during negative versus positive videos, pointing to the distinct impact of the valence of the videos on participants' feelings of empathy versus their levels of empathic accuracy. Hence, feeling empathy and being empathically accurate in inferencing what others might feel is not the same.

A signalling function of eye gaze has been previously mentioned in the literature (Cowan et al., 2014; Kobayashi & Hashiya, 2011; Mason et al., 2005), although empirical evidence was lacking. Kobayashi and Hashiya (2011), for example, introduced the “gaze‐grooming” hypotheses, stating that gaze has evolved into a contact‐free, social grooming function in humans to form and maintain social bonds. Our results are in line with this “gaze‐grooming” hypotheses, and the various target stories deriving from distinct targets show empirical evidence for the generalizability of this signalling function of gaze to a variety of social situations.

This study uniquely examined to what extent gazing at the eye region of others contributes to participants' EA under ecologically valid circumstances. The methodological design of the EA task not only allows for a corresponding assessment of the feelings of both perceiver and target in positive and negative situations, but also incorporates the assessment of fluctuations in their affect over time. Furthermore, the novel addition of individual ratings about perceivers' affect and state empathy after each video informed us on how participants subjectively experienced the emotional target stories and gives additional insight in the validity of the task.

While the richness of the dynamic stimuli, including both verbal and non‐verbal information, are a major advantage of the present study, future studies could focus on the individual contribution of the verbal and visual content to EA (Zaki et al., 2009). As perceivers were presented with videos of unknown targets, they were well aware that they were not involved in an actual bidirectional conversation. This may have lowered their motivation to be empathically accurate and may have affected our findings. Related to this, the videos do not mimic bidirectional interactions, but rather mimic listening to a monologue. It is important to mention that these are two different types of interactions that occur under different circumstances. As the EA task more closely mimics the latter, our findings are probably most generalizable to closely resembling situations in real‐life, such as (mental) health settings in which practitioners are listening to personal stories of their clients. Furthermore, participants were placed in a chin rest while watching the videos to limit head motion. Although they reported low irritability during the task and EA levels were comparable to prior studies using the EA task, it is possible that they experienced the chin rest as unpleasant, which might have affected their performances. Lastly, it is of note that the participants in this study are adults aged between 35 and 64 years (*M* = 48; *SD* = 5.50) and the results of the current study need to be interpreted in the context of this age group.

## CONCLUSION

While prior studies have shown the importance of the eye region for inferring others' feelings when only visual information is available, our results show that gazing to the eyes of others may not contribute to EA when both visual and verbal information is available. In addition, gazing to the eyes of others seem to be a way to express empathy and social engagement to others. In other words, our results inform us on the role of eye gazing during social interactions and shed light on a possible signalling function of eye gazing to sympathize or empathize with our conversational partners.

This outcome, compared to that obtained using less ecologically valid paradigms, emphasises the importance of studying how individuals perceive others in social settings that closely mimic real‐life. Our findings enrich the field of social sciences in several ways and implicate that we need to be very careful in translating findings from basic science to the complex realm of daily life. On a theoretical level, there is a clear need to better understand the factors that contribute to EA in daily life, as our data seem to suggest that gazing to the eyes is not a substantial source of information in our daily conversations. At the methodological level, these results make us aware of the way methodological differences between studies give rise to diverging outcomes and that a combination of both basic experiments and designs including more ecologically valid measures is needed to better understand social interactions. Last, our findings may have implications at the practical level for communication between people in general, and might be of particular relevance for (mental) health care practitioners in medical or therapeutic settings. Signalling their empathy and emotional engagement by gazing to the eyes of their clients, especially when listening to their (emotionally valenced) personal stories, might be particularly helpful in favouring the quality of the therapeutic relationship.

## Supporting information


**Appendix S1** Supporting Information

## Endnotes

^1^
After the lab visit, families were (a) asked to fill out ecological momentary assessments for 14 consecutive days on their smartphones, using a mobile app to assess affect and parent‐adolescent interactions in daily life, and (b) invited for an MRI session on a separate day. Data derived from these parts of the RE‐PAIR study are reported elsewhere.
