# Supplementary material for: Eyes on you: Ensuring empathic accuracy or signalling empathy?
Source: Int J Psychol. 2022 Jun 13;57(6):743–52. doi: 10.1002/ijop.12862 (PMC9796408; doi:10.1002/ijop.12862)
Supplement: Supplementary file 1 — Appendix S1 Supporting Information [file IJOP-57-743-s001.docx]

**SUPPLEMENTARY MATERIALS**

**SUPPLEMENT 1**

Inclusion criteria were applied to the adolescent and included being aged between 11 and 17 years, and living with at least one of their parents. All adolescents were screened on current and lifetime psychopathology with the Kiddie-Schedule for Affective Disorders and Schizophrenia-Present and Lifetime Version (K-SADS PL; Kaufman, Birmaher, Brent, Rao, and Ryan (1996)). Adolescents without psychopathology and their parents were included in the study in case the adolescents did not meet criteria for any (neuro)psychiatric disorder in the two years leading up to the study, and had no lifetime diagnoses of MDD/dysthymia. Adolescents with psychopathology and their parents were included in case the adolescent met criteria for a current, primary diagnosis of MDD/dysthymia according to the K-SADS.

**SUPPLEMENT 2**

The present study includes a shortened version of the EA task with an approximate duration of 25 minutes instead of the 50 minutes of the original version of the task. The subset of videos was derived from a pilot study in a student sample (*n* = 20) in which we tested the feasibility of the already existing EA task in combination with an eye tracking set-up. Videos were selected based on their feasibility for eye tracking purposes (i.e., videos in which targets were excessively moving were excluded) and sufficient variety in EA ratings per video in the pilot study (range mean EA between 0.35-0.85; SD > 0.30). Also, we tested two versions of the task in which one version contained the presentation of the 9-point Likert scale of the dial below the video on the screen (similar to the original task), while the other version did not include the presentation of this scale on the screen. The data of the pilot indicated that participants often looked at the scale on the screen instead of the video, which was not desirable for the eye tracking measures. We therefore decided to use the version without the scale on the screen for the present study.

**SUPPLEMENT 3**

A customized MATLAB script (MathWorks, Inc., Natick, MA, version 9.5) was used to pre-process raw eye tracking data into measures of eye gaze per perceiver per video. Raw gaze data of at least one eye was used to calculate information of gaze position and duration. Furthermore, validity of gaze data was calculated as the percentage of successfully recorded eye tracking data per video as an estimate of data quality. Individual videos of which the validity was below 70% were excluded from analyses. In order to follow the natural movement of the targets in the videos, dynamically moving areas of interest (AOI) were created around the left eye, right eye, mouth, and face as a whole of all individual targets using MATLABs cascade object detector, which uses the algorithm of Viola and Jones (2001) for face and facial feature detection. Specifically, for each frame of each video, this algorithm outputted rectangle AOIs encompassing the left eye, right eye, mouth, and face (see below). Outlier removal, smoothing, and interpolation was performed on the AOIs thereafter, to correct any incorrectly identified AOIs due to movement or blinking of the target in the video. The gaze data within the right and left eye AOIs were corrected for overlap and combined into a single AOI for the eye region. The screenshot of the target person presented below is blurred due to privacy reasons.


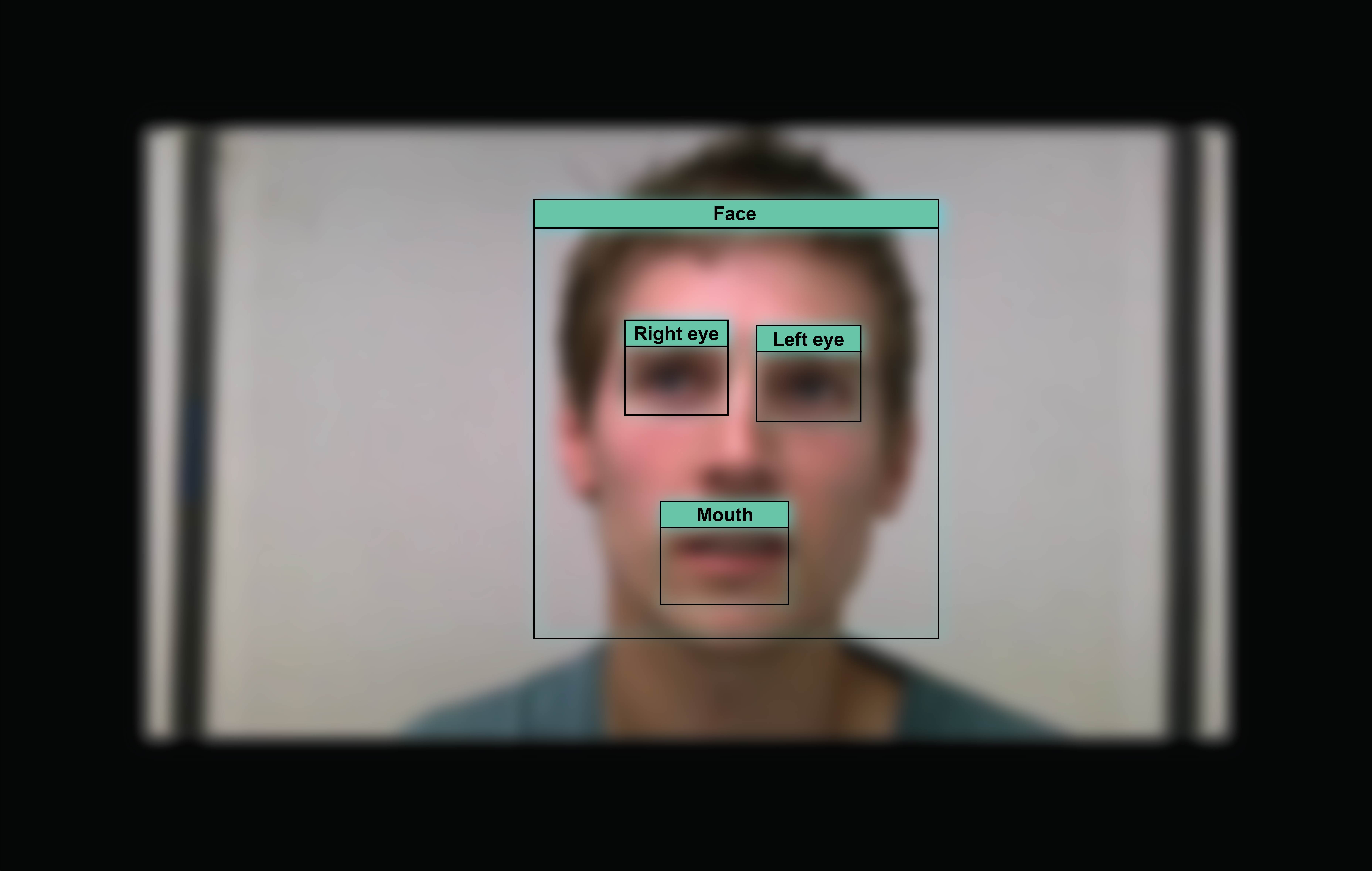


**SUPPLEMENT 4**

Empathic accuracy and gaze data for positive and negative videos.

|  | All videos | Positive videos | Negative videos | Differences in valence^1^ | |
| --- | --- | --- | --- | --- | --- |
| Mean (SD) | *n* = 981 | *n* = 486 | *n* = 495 | *t* | *p* |
| *EA (Fisher z transformed)* | | | | | |
| All | 0.84 (0.40) | 1.09 (0.45) | 0.63 (0.51) | 8.25 | <0.001 |
| Males | 0.79 (0.44) | 1.03 (0.51) | 0.53 (0.53) | 5.52 | <0.001 |
| Females | 0.93 (0.35) | 1.15 (0.40) | 0.70 (0.49) | 6.13 | <0.001 |
| *Dwell time face AOI, %* | | | | | |
| All | 85.70 (8.65) | 85.34 (8.79) | 86.05 (8.74) | -2.43 | 0.015 |
| Males | 85.56 (9.04) | 84.97 (9.29) | 86.15 (9.02) | -2.67 | 0.007 |
| Females | 85.81 (8.40) | 85.64 (8.42) | 85.96 (8.57) | -0.91 | 0.363 |
| *Dwell time eyes AOI, %* | | | | | |
| All | 33.38 (18.49) | 31.39 (17.76) | 35.39 (19.67) | -6.15 | <0.001 |
| Males | 32.60 (20.69) | 30.60 (20.03) | 34.59 (21.73) | -4.49 | <0.001 |
| Females | 34.02 (16.65) | 32.02 (15.82) | 36.04 (17.99) | -4.22 | <0.001 |
| *Dwell time mouth AOI, %* | | | | | |
| All | 15.79 (15.14) | 15.58 (14.86) | 16.02 (15.56) | -1.18 | 0.240 |
| Males | 17.40 (17.00) | 17.08 (16.74) | 17.74 (17.35) | -0.57 | 0.569 |
| Females | 14.48 (13.45) | 14.36 (13.15) | 14.62 (13.93) | -1.07 | 0.287 |
| *Individual state empathy and affect ratings after each video* | | | | | |
| Empathic concern | 5.05 (1.44) | 4.95 (1.42) | 5.14 (1.47) | -2.52 | 0.012 |
| Perspective taking | 5.19 (1.41) | 5.11 (1.42) | 5.27 (1.39) | -2.13 | 0.033 |
| Happy | 4.11 (1.60) | 4.92 (1.30) | 3.28 (1.45) | 22.13 | <0.001 |
| Sad | 2.25 (1.56) | 1.56 (1.00) | 2.96 (1.71) | -18.89 | <0.001 |
| Irritated | 1.61 (1.13) | 1.59 (1.14) | 1.63 (1.13) | -0.91 | 0.366 |
| Relaxed | 5.06 (1.45) | 5.27 (1.39) | 4.83 (1.47) | 7.17 | <0.001 |

*Note.* SD, standard deviation; EA, empathic accuracy; AOI, area of interest.

^1^ Differences in valence were calculated by generalized linear mixed models and were tested without covariates in the model.

**SUPPLEMENT 5**

EA task data perceivers per video.

|  | | | | Perceiver ratings per video, mean (SD) | | | | | | |
| --- | --- | --- | --- | --- | --- | --- | --- | --- | --- | --- |
| Videos | Duration (s) | Valence | *n* | EA _Fisher_ *_z_* _transformed_ | Empathic concern | Perspective taking | Happiness | Sadness | Relaxed | Irritated |
| 1 | 116 | N | 98 | 1.15 (1.33) | 5.59 (1.38) | 5.72 (1.31) | 2.60 (1.33) | 3.61 (1.78) | 4.57 (1.60) | 1.46 (0.98) |
| 2 | 83 | P | 97 | 1.17 (0.59) | 4.59 (1.43) | 4.75 (1.51) | 4.60 (1.27) | 1.59 (1.06) | 5.32 (1.26) | 1.58 (1.13) |
| 3 | 107 | N | 95 | 0.56 (1.34) | 4.66 (1.56) | 4.91 (1.50) | 3.48 (1.30) | 2.72 (1.48) | 4.97 (1.35) | 1.80 (1.37) |
| 4 | 104 | N | 96 | 0.30 (0.62) | 4.74 (1.39) | 4.84 (1.33) | 3.93 (1.34) | 2.36 (1.40) | 4.94 (1.47) | 1.61 (0.96) |
| 5 | 155 | N | 101 | 0.66 (0.46) | 5.68 (1.27) | 5.69 (1.22) | 2.55 (1.35) | 3.71 (1.82) | 4.61 (1.48) | 1.74 (1.35) |
| 6 | 103 | P | 99 | 0.55 (0.90) | 4.87 (1.46) | 5.07 (1.47) | 4.78 (1.37) | 1.78 (1.15) | 5.29 (1.28) | 1.86 (1.30) |
| 7 | 102 | P | 99 | 1.41 (1.09) | 4.62 (1.49) | 4.79 (1.43) | 4.55 (1.43) | 1.70 (1.08) | 5.19 (1.46) | 1.81 (1.33) |
| 8 | 105 | P | 100 | 1.11 (0.80) | 4.95 (1.31) | 5.14 (1.33) | 5.06 (1.13) | 1.49 (0.89) | 5.20 (1.44) | 1.45 (1.06) |
| 9 | 121 | N | 96 | 0.48 (0.41) | 4.99 (1.44) | 5.16 (1.34) | 3.88 (1.28) | 2.32 (1.46) | 5.09 (1.38) | 1.54 (0.92) |
| 10 | 109 | P | 100 | 1.20 (0.49) | 5.72 (1.08) | 5.80 (1.11) | 5.62 (0.97) | 1.26 (0.68) | 5.37 (1.52) | 1.24 (0.62) |

*Note.* S, Seconds; EA, Empathic accuracy; N, negative video; P, positive video.

**SUPPLEMENT 6**

Generalized linear mixed regression model to assess the influence of valence, target expressivity, and empathic concern and perspective taking of perceivers on the level of EA (Fisher *z* transformed).

|  | *b* | SE | df | *t* | *p* |
| --- | --- | --- | --- | --- | --- |
| **Model 1** |  |  |  |  |  |
| *Intercept^1^* | 0.630 | 0.048 | 235.6 | 13.22 | <0.001 |
| Valence_positive_ | 0.457 | 0.055 | 881.9 | 8.25 | <0.001 |
| **Model 2** |  |  |  |  |  |
| *Intercept^1^* | -0.915 | 0.159 | 959.7 | 5.75 | <0.001 |
| BEQ | -0.013 | 0.037 | 883.7 | -0.35 | 0.730 |
| **Model 3** |  |  |  |  |  |
| *Intercept^1^* | 0.812 | 0.148 | 104.4 | 5.48 | <0.001 |
| Empathic concern | 0.003 | 0.008 | 103.5 | 0.34 | 0.733 |
| **Model 4** |  |  |  |  |  |
| *Intercept^1^* | 0.822 | 0.155 | 106.8 | 5.30 | <0.001 |
| Perspective taking | 0.002 | 0.009 | 106.7 | 0.26 | 0.796 |

^1^ The intercept includes perceivers’ level of empathic accuracy (Fisher *z* transformed) during negative autobiographical stories.

**SUPPLEMENT 7**

Perceivers’ average percentage of dwell time in all AOIs per video.

|  |  |  |  | % dwell time per AOI, mean (SD) | | |
| --- | --- | --- | --- | --- | --- | --- |
| Videos | Duration (s) | Valence | *n* | Eyes | Mouth | Face |
| 1 | 116 | N | 98 | 33.01 (21.96) | 15.64 (16.33) | 86.09 (9.46) |
| 2 | 83 | P | 97 | 30.90 (19.96) | 15.09 (15.53) | 85.40 (10.02) |
| 3 | 107 | N | 95 | 34.97 (21.72) | 16.90 (16.17) | 87.29 (8.78) |
| 4 | 104 | N | 96 | 35.51 (20.67) | 17.42 (16.68) | 87.38 (8.41) |
| 5 | 155 | N | 101 | 43.96 (22.30) | 15.54 (14.99) | 85.89 (8.95) |
| 6 | 103 | P | 99 | 42.71 (22.23) | 17.16 (17.26) | 85.25 (8.50) |
| 7 | 102 | P | 99 | 31.49 (17.74) | 17.50 (14.84) | 86.90 (8.43) |
| 8 | 105 | P | 100 | 28.49 (16.50) | 12.13 (14.00) | 86.52 (8.74) |
| 9 | 121 | N | 96 | 33.89 (19.76) | 15.88 (16.91) | 87.77 (8.35) |
| 10 | 109 | P | 100 | 29.12 (17.96) | 16.76 (16.51) | 86.89 (8.01) |

*Note.* AOI, area of interest; s, seconds; N, negative video; P, positive video.

**SUPPLEMENT 8**

EA task data targets per video.

|  |  |  |  | Targets | | | |
| --- | --- | --- | --- | --- | --- | --- | --- |
| Videos | Duration (s) | Valence | *n* | Target nr. | BEQ | Age | Gender |
| 1 | 116 | N | 98 | 1 | 3.5 | 25 | Male |
| 2 | 83 | P | 97 | 1 | 3.5 | 25 | Male |
| 3 | 107 | N | 95 | 2 | 5.03 | 26 | Female |
| 4 | 104 | N | 96 | 2 | 5.03 | 26 | Female |
| 5 | 155 | N | 101 | 3 | 4.06 | 62 | Male |
| 6 | 103 | P | 99 | 3 | 4.06 | 62 | Male |
| 7 | 102 | P | 99 | 4 | 5.97 | 24 | Female |
| 8 | 105 | P | 100 | 5 | 4.03 | 26 | Male |
| 9 | 121 | N | 96 | 6 | 3.61 | 23 | Female |
| 10 | 109 | P | 100 | 6 | 3.61 | 23 | Female |

*Note.* BEQ, Berkeley Expressivity Scale

**SUPPLEMENT 9**

**Average levels of the percentage missing gaze data as part of the total video duration per video order as presented in the task.**

The percentage missing gaze data did not differ between the videos, indicating that there was not more missing gaze data at the start of the task compared to later moments in the task. This indicates that the missing gaze data did not depend on the time point within the task. Error bars represent standard errors of the mean.

**
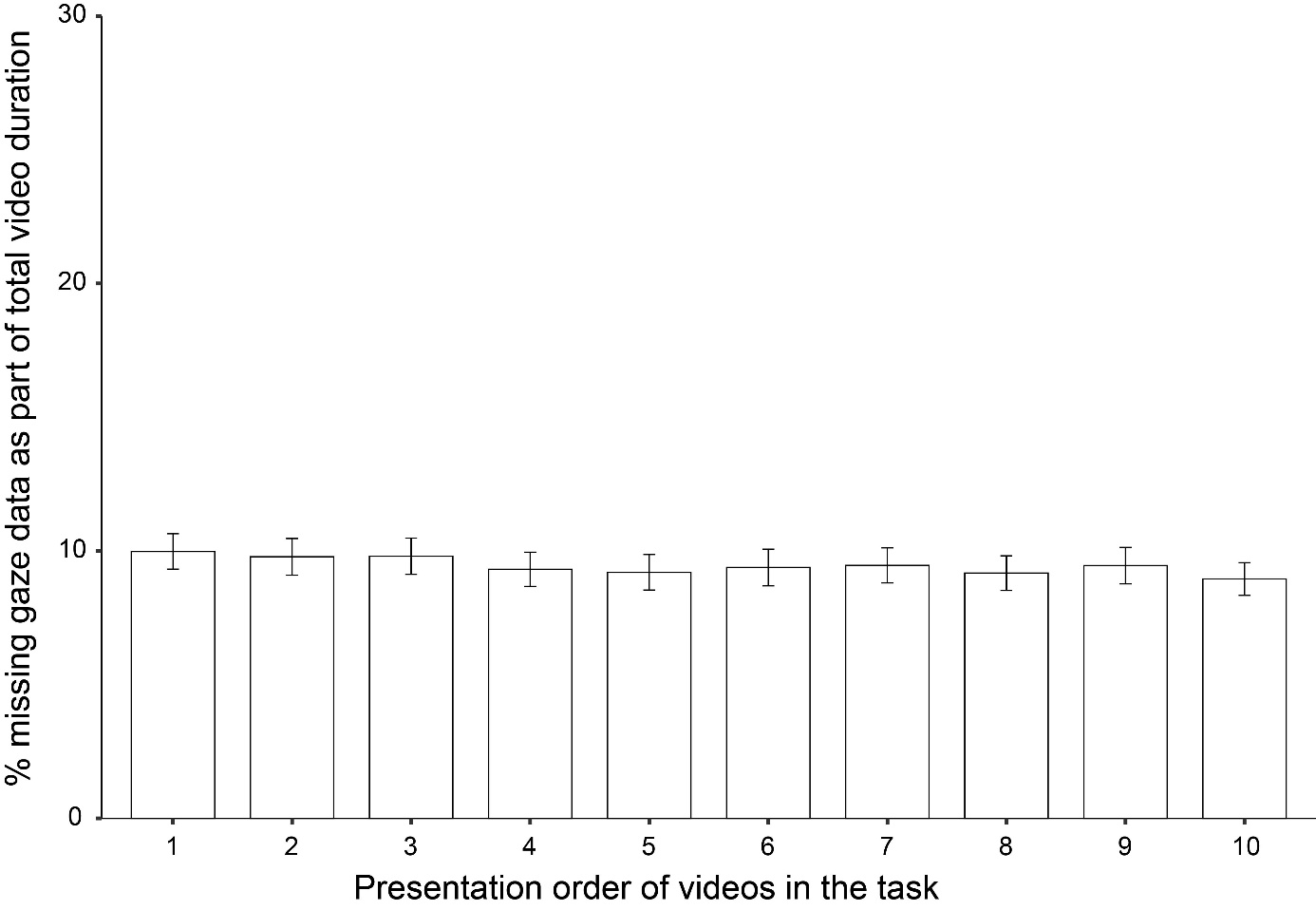
**

**SUPPLEMENT 10**

Generalized linear mixed regression model to assess the main effects and interaction of valence (positive versus negative) and target expressivity (BEQ average scores) on perceivers’ level of EA (Fisher *z* transformed).

|  | *b* | SE | df | *t* | *p* |
| --- | --- | --- | --- | --- | --- |
| **Model 1** |  |  |  |  |  |
| *Intercept^1^* | 0.948 | 0.071 | 173.4 | 13.29 | <0.001 |
| % dwell time eye region | -0.003 | 0.002 | 212.2 | -1.46 | 0.146 |
| **Model 2** |  |  |  |  |  |
| *Intercept^1^* | 0.522 | 0.090 | 389.0 | 5.81 | <0.001 |
| % dwell time eye region | 0.003 | 0.002 | 460.4 | 1.41 | 0.158 |
| Valence_positive_ | 0.761 | 0.108 | 888.5 | 7.07 | <0.001 |
| % dwell time eye region * Valence_positive_ | -0.009 | 0.003 | 892.8 | -3.33 | <0.001 |
| **Model 3** |  |  |  |  |  |
| *Intercept^1^* | 1.209 | 0.309 | 937.7 | 3.92 | <0.001 |
| % dwell time eye region | -0.009 | 0.008 | 924.8 | -1.11 | 0.269 |
| BEQ | -0.062 | 0.072 | 895.3 | -0.87 | 0.385 |
| % dwell time eye region * BEQ | 0.002 | 0.002 | 895.6 | 0.81 | 0.416 |
| **Model 4** |  |  |  |  |  |
| *Intercept^1^* | 2.085 | 0.481 | 905.7 | 4.33 | <0.001 |
| % dwell time eye region | -0.008 | 0.012 | 899.3 | -0.70 | 0.483 |
| Valence_positive_ | -1.105 | 0.602 | 885.3 | -1.84 | 0.067 |
| BEQ | -0.370 | 0.112 | 887.8 | -3.31 | <0.001 |
| % dwell time eye region * Valence_positive_ | -0.006 | 0.016 | 884.5 | -0.37 | 0.711 |
| % dwell time eye region * BEQ | 0.003 | 0.003 | 887.6 | 0.97 | 0.331 |
| Valence_positive_ * BEQ | 0.441 | 0.141 | 885.6 | 3.13 | 0.002 |
| % dwell time eye region * Valence_positive_ * BEQ | -0.001 | 0.004 | 885.1 | -0.21 | 0.833 |

^1^ The intercept includes perceivers’ level of EA (Fisher *z* transformed) during negative autobiographical target stories.

***References***

Kaufman, J., Birmaher, B., Brent, D., Rao, U., & Ryan, N. (1996). Kiddie-Sads-present and Lifetime version (K-SADS-PL). *Pittsburgh, University of Pittsburgh, School of Medicine.* doi:10.1097/00004583-199707000-00021
